# Coronary Collateral Circulation in Coronary Syndromes: Echocardiographic Insights and Future Perspectives

**DOI:** 10.3390/diagnostics16213694

**Published:** 2026-11-09

**Authors:** Diana Zamfir, Alexandra Ioana Dumitrache, Diana Irena Cojocaru, Alexandru Scafa-Udriște

**Affiliations:** 1 Department of Cardiology, Clinical Hospital CF2 Bucharest, University of Medicine and Pharmacy Carol Davila, 050474 Bucharest, Romania; alexandru.scafa@umfcd.ro; 2 Department of Cardiology, 4th Clinical Department–Cardiothoracic Pathology, University of Medicine and Pharmacy Carol Davila, 050474 Bucharest, Romania; alexandra-ioana-floriana.dumitrache@drd.umfcd.ro; 3 Department of Cardiology, Emergency Clinical Hospital Bucharest, University of Medicine and Pharmacy “Carol Davila”, 050474 Bucharest, Romania; diana-irena.stanciulescu@drd.umfcd.ro

**Keywords:** coronary collateral circulation, echocardiography, speckle tracking, myocardial work, stress echocardiography, myocardial contrast echocardiography, artificial intelligence

## Abstract

Coronary collateral circulation (CCC) represents a natural adaptive mechanism capable of preserving myocardial perfusion in the presence of obstructive coronary artery disease. Although invasive angiography remains the reference method for collateral visualization, it provides limited information regarding collateral functionality and downstream myocardial protection. Echocardiography, due to its widespread availability, lack of radiation, and advances in imaging techniques, has emerged as an attractive non-invasive tool for evaluating the myocardial consequences of collateral perfusion. This review summarizes current evidence regarding the role of echocardiography in assessing coronary collateral circulation. Conventional echocardiographic parameters, stress echocardiography, myocardial contrast echocardiography, speckle-tracking imaging, myocardial work analysis and three-dimensional echocardiography are reviewed in the context of collateral-dependent myocardial function. Emerging technologies including artificial intelligence and quantitative perfusion imaging are also discussed. Rather than directly visualizing collateral vessels, echocardiography provides complementary information on the myocardial consequences of collateral-dependent perfusion through assessment of regional function, myocardial mechanics, and, with contrast techniques, tissue perfusion. However, collateral-specific validation remains limited, particularly for emerging deformation and myocardial work indices, and echocardiographic findings should be interpreted alongside coronary anatomy and physiological or perfusion measurements. Future integration with invasive assessment, multimodality imaging, and molecular markers may improve characterization of collateral-dependent myocardium.

## 1. Introduction

Coronary collateral circulation (CCC) consists of a network of pre-existing intercoronary vascular connections that can open, respectively enlarge in response to acute or progressive coronary artery obstruction. Through arteriogenesis and functional recruitment, these vessels provide an alternative source of blood flow to myocardium jeopardized by a critically stenosed or occluded epicardial coronary artery in acute as well as chronic coronary artery disease (CAD). However, the extent and functional capacity of collateralization vary considerably among individuals and cannot be reliably predicted solely from the anatomical severity or duration of coronary artery disease (CAD). Well-developed collaterals may limit myocardial ischemia, reduce infarct size, preserve ventricular function, and attenuate adverse remodeling in both acute and chronic coronary syndromes [1]. Their clinical importance is further supported by evidence associating a high degree of collateralization with a significant reduction in all-cause mortality among patients with CAD [2].

The evaluation of coronary collaterals remains challenging. Invasive coronary angiography is the most frequently used method for collateral visualization, introducing semiquantitative angiographic scores such as the Rentrop classification. Nevertheless, angiography primarily describes collateral anatomy and provides limited information regarding the amount of blood flow delivered to the collateral-dependent myocardium. Pressure- or Doppler-derived collateral flow index offers a more objective functional assessment and remains the invasive reference standard; however, it requires coronary instrumentation and temporary vessel occlusion, restricting its routine clinical use [1,3].

Echocardiography is a widely available, radiation-free, and repeatable investigation for evaluating the myocardial consequences of collateral perfusion. Rather than directly visualizing the small and anatomically complex collateral vessels, conventional and advanced echocardiographic techniques assess their functional effectiveness through regional wall motion, contractile reserve, myocardial perfusion and ventricular remodeling. Myocardial contrast echocardiography is particularly relevant because it can quantify microvascular blood flow within collateral-dependent territories and has demonstrated agreement with invasively measured collateral function [3,4]. Stress echocardiography, speckle-tracking imaging, and newer myocardial work indices may offer additional information about the capacity of collateral flow to preserve myocardial mechanics under resting and ischemic conditions. This narrative review critically synthesizes the evidence for conventional and advanced echocardiographic assessment of collateral-dependent myocardium across acute and chronic coronary syndromes. Its specific contribution is to distinguish collateral anatomy and physiology from downstream perfusion and mechanical consequences, compare the principal supporting studies, and identify the validation gaps that currently limit clinical application.

### Literature Search and Selection

This narrative review was informed by searches of PubMed/MEDLINE, Web of Science, and Scopus from database inception to 30 September 2026. Search terms included combinations of “coronary collateral circulation,” “collateral flow index,” “echocardiography,” “stress echocardiography,” “myocardial contrast echocardiography,” “speckle tracking,” “myocardial strain,” and “myocardial work.” Publications were selected according to their relevance to collateral physiology, echocardiographic assessment of collateral-dependent myocardial perfusion and function, and complementary imaging methods. Original clinical and physiological studies were considered alongside relevant mechanistic studies, reviews, and professional recommendations. Findings were synthesized qualitatively, considering study design, clinical setting, collateral assessment methods, and principal limitations. The review was intended to provide a focused narrative synthesis rather than an exhaustive systematic assessment.

## 2. Coronary Collateral Circulation: Anatomy and Pathophysiological Background

### 2.1. Anatomy, Native Collateral Vessels, Collateral Circulation

Knowing the anatomy of coronary circulation and variants is critical in clinical and interventional cardiology as well as in cardiothoracic surgery. The typical layout of coronary anatomy is represented by the two main arteries and their branches: left main coronary artery, which originates from the left aortic sinus of Valsalva and the right coronary artery, which originates form the right aortic sinus. Coronary collaterals can be intercoronary (between two coronary arterial systems, such as left anterior descending artery to right coronary artery) or intracoronary (between different branches of the same coronary artery). The importance of coronary collateral circulation is still being thoroughly studied. In patients with coronary artery disease, ischemia-protective collaterals can be found in about 33% and their presence is directly linked to survival improvement [1].

### 2.2. Angiogenesis and Arteriogenesis

Coronary collaterals are naturally developed inter-arterial anastomoses that connect different major coronary arteries or their branches. Under baseline conditions with normal coronary perfusion, these micro-vessels are usually closed or carry minimal flow. Conversely, in chronic or acute reductionblood flow due to arterial occlusion or severe stenosis, pressure gradients and hypoxia stimulate collateral pathways to dilate, increasing blood flow to the ischemic myocardium [5]. At the present time, angiogenesis is the process through which these capillary networks expand and remodel into a mature vascular system. Moreover, arteriogenesis represents the transformation of arterioles into muscular collateral arteries through a more complex process that includes smooth muscle proliferation [1]. Enhancing arteriogenesis in such patients may represent a promising non-invasive therapy.

### 2.3. Molecular Regulation

During angiogenesis, new capillary structures are formed from pre-existing vascular networks and the structural maturation of these vessels requires recruitment of pericytes, endothelial cells, vascular smooth muscle cells. Tissue hypoxia upregulates hypoxia-inducible Factor 1-alpha (HIF-1α) and increases endothelial cell sensitivity to pro-angiogenic factors through up-regulation of their cell-surface receptors [6,7].

Moreover, this process is sustained by local inflammatory signaling. Infiltrating macrophages and monocytes release matrix metalloproteinases (MMPs) due to the degradation of the extracellular matrix, allowing endothelial cells to detach and migrate to form new vessels.

The preclinical success of administration of angiogenic growth factors as vascular endothelial growth factor (VEGF) or basic fibroblast growth factor (bFGF) showed clear improvements in collateral function in animal models, whereas in human trials their efficacy is yet to be proven in controlled clinical trials [8].

In ischemic condition, when there is a drop in post-stenotic pressure, the blood flow is redistributed to pre-existing arterioles and the longitudinal shear stress on endothelium is increased in those areas, leading to endothelial activation through the up-regulation of chemokines and adhesion molecules [9].

Arteriogenesis has been proven to be induced by monocyte chemotactic protein-1 (MCP-1), tumor necrosis factor alpha (TNF-α), FGF and lipopolysaccharide, activated macrophages and granulocyte-macrophage colony stimulating factor (recombinant human GM-CSF) [10].

The local inflammation leads to monocyte recruitment and infiltration of the tissue, followed by smooth muscle cell proliferation and outward structural remodeling. The result is obtaining large and functional bridging collaterals [1].

One important contribution in adaptive vascular growth is that of microRNAs (ribonucleic acids), important regulators of gene expression in a wide range of organisms and biological processes, which have been found to be involved in almost all the stages of neovascularization: basic signaling through miR-100, cell–matrix and cell–cell interactions (miR-92a) and angiogenic cytokine secretion (miR-93). MicroRNA based therapy could be a major step in therapy of cardiovascular disease [11].

## 3. Clinical Impact

Collateral function is a direct indicator of coronary artery disease severity which can indicate a poor outcome in coronary artery disease. However, it is widely known that their myocardial salvaging effect on a myocardial area jeopardized by ischemia [1].

In patients with acute myocardial infarction, well-developed coronary collaterals have been associated with smaller infarct size. In a meta-analysis of six studies involving 820 patients, Best et al. found significantly larger CMR-derived infarct size in patients with poor versus good collaterals. However, the pooled difference in LVEF was not statistically significant [12]. These findings support an association between collateral development and reduced myocardial injury but do not, by themselves, establish improved survival or a significant difference in LVEF.

Nathoe et al. evaluated the prognostic significance of angiographically visible collaterals in 561 patients enrolled in a randomized trial comparing coronary stenting with coronary artery bypass grafting. Collaterals, defined as Rentrop grade >1, were present in 176 patients (31%) and were associated with lower odds of cardiac death or myocardial infarction at 1 year after revascularization (adjusted odds ratio 0.18; 95% confidence interval 0.04–0.78) Although the revascularization strategy was randomized, the comparison according to collateral status was observational and therefore remains susceptible to residual confounding.

Koerselman et al. examined the prognostic significance of angiographically visible coronary collaterals in a prospective case–cohort study of 655 patients admitted for elective coronary angioplasty. Collateral presence was associated with an unfavorable prognosis, particularly in higher-risk patients [13]. In contrast to the protective association reported by Nathoe et al. [14]. these findings illustrate the difficulty of separating collateral-mediated protection from the underlying coronary disease burden. Angiographic collateralization may therefore represent both a compensatory response to ischemia and a marker of advanced disease, rather than an independent indicator of favorable or unfavorable outcomes.

## 4. Current Methods for Assessing Coronary Collaterals

Coronary collaterals should be assessed as a functionally relevant collateral circulation, rather than solely as angiographically visible vessels. Angiography depicts donor-to-recipient filling, whereas the clinically important question is whether collateral flow preserves perfusion in the jeopardized myocardial territory—particularly under stress. This distinction matters because angiographic anatomy does not reliably represent functional collateral perfusion [15,16].

### 4.1. Invasive Assessment

The Rentrop score is the conventional angiographic, semiquantitative assessment. During contrast injection into the donor artery, grade 0 denotes no visible collateral filling; grade 1, filling of side branches of the occluded artery without visualization of the epicardial segment; grade 2, partial epicardial filling; and grade 3, complete epicardial filling. Advantages are speed, familiarity, no need for dedicated pressure or Doppler hardware, and immediate applicability during diagnostic angiography or percutaneous coronary intervention PCI [17]. Its central limitation is that it measures contrast opacification rather than myocardial blood flow, also collaterals below angiographic resolution can be functionally relevant. Thus, Rentrop grading is useful for anatomical description and communication, but it should not be interpreted as a quantitative measure of collateral reserve or as a stand-alone viability test [4,16].

Collateral flow index (CFI) is the principal invasive quantitative measure of collateral function. It expresses collateral flow during transient coronary occlusion as a fraction of antegrade flow when the recipient artery is patent. It can be calculated from distal coronary pressure (pressure-derived CFI) or from Doppler-derived flow velocity (velocity-derived CFI); the underlying conceptual formulation is:

CFI_p_ = P_occl_ − P_CV_/P_ao_ − P_CV_

where P_occl_ is distal coronary occlusive pressure, P_ao_ is aortic pressure, and P_CV_ is central venous pressure. Pressure- and Doppler-derived indices have shown good agreement and both provide a physiological rather than purely anatomical estimate [17,18]. The advantages of CFI are its quantitative scale, ability to detect recruitable collaterals not seen on angiography, and direct relationship to ischemia during balloon occlusion. In one PTCA study, patients without ECG signs of ischemia during occlusion had average relative collateral flow of 46%, versus 18% among those with ischemia; a 30% CFI threshold discriminated the two groups well in that cohort [18]. Its limitations are equally important: CFI requires invasive instrumentation and a transient, controlled occlusion; it is therefore most feasible during PCI rather than routine diagnostic assessment. In chronic total occlusion, pressure/Doppler assessment also requires successful passage of the sensor through the true lumen, which can be impossible before recanalization [16].

A pressure wire positioned distal to the stenosis or occlusion measures distal coronary/wedge pressure during balloon occlusion, alongside aortic and central venous pressure, to derive. Relative to Doppler, it is generally the more practical invasive technique because pressure acquisition is technically straightforward and less dependent on signal-quality optimization. It provides a reproducible physiologic endpoint and can be integrated into a PCI procedure [17,18].

However, pressure-derived CFI is still an occlusion-dependent measurement. It reflects the net pressure support delivered through collaterals, not direct tissue perfusion, and may be influenced by venous pressure, distal microvascular resistance, and the duration and hemodynamic stability of occlusion. It also adds procedure time, cost, and the risks inherent to coronary wiring [16,17].

A Doppler guidewire measures collateral flow velocity distal to the occluded segment during balloon occlusion and permits the calculation of velocity-derived CFI. Its particular advantage is direct characterization of the velocity waveform and flow direction. In the Doppler study of 43 patients, longer-duration and unidirectional collateral-flow patterns were associated with fewer ECG signs of ischemia [19]. Doppler CFI is more technically demanding than pressure measurement: obtaining and maintaining an adequate velocity signal depends on wire position and alignment, and the method samples velocity rather than absolute volumetric flow. Like pressure-wire assessment, it requires temporary occlusion and is not suitable for routine serial evaluation. Nevertheless, Doppler-derived CFI and pressure-derived CFI were closely correlated in a PCI cohort, supporting their use as complementary invasive physiological approaches [18].

### 4.2. Non-Invasive Assessment

No non-invasive modality directly reproduces wire-based CFI. Their role is different but clinically valuable: they identify the downstream consequence of collateral supply—resting perfusion, inducible ischemia, flow reserve, scar, and viability—without coronary instrumentation. A collateral network can meet resting metabolic demand but can fail during stress; stress-perfusion imaging is therefore more informative than resting imaging alone when the question is functional adequacy [4,16].

Coronary CT angiography (CCTA) provides high-resolution anatomical assessment of coronary stenosis/occlusion, calcium, plaque, and major visible collateral channels. It is useful for defining the coronary substrate in which collaterals have developed. Dynamic or stress CT perfusion can add a tissue-perfusion readout, but CT does not routinely quantify collateral flow itself. Its main disadvantages are ionizing radiation, iodinated contrast, heart-rate/motion and calcium-related image-quality limitations, and less established collateral-specific validation than invasive CFI or quantitative perfusion imaging [15,20].

Stress perfusion cardiac magnetic resonance (CMR) evaluates inducible regional hypoperfusion; late gadolinium enhancement characterizes infarction/scar, while cine imaging assesses ventricular function. Consequently, CMR is particularly helpful when the clinical question is whether collateralized myocardium remains viable and functionally protected rather than whether a collateral vessel is angiographically visible. Contemporary reviews place quantitative CMR perfusion alongside positron emission tomography PET as a function-first approach to collateral assessment [15]. CMR offers excellent tissue characterization and no ionizing radiation, but availability, scan duration, patient tolerance, device compatibility, arrhythmia, and gadolinium-related constraints can limit use. It measures the myocardial consequence of collateral supply, not CFI directly [15,20].

PET myocardial perfusion imaging can quantify absolute myocardial blood flow and flow reserve, making it a strong non-invasive option for determining whether an apparently collateralized territory has preserved stress perfusion. PET and CMR had better pooled diagnostic performance for hemodynamically significant CAD than SPECT in a 2021 meta-analysis, although that comparison addresses CAD detection rather than direct validation of collateral flow [21]. The strengths of PET are quantitative perfusion assessment and high image quality; the limitations are limited availability, cost, tracer logistics, and radiation exposure. For collateral research, PET is best considered as a measure of tissue-level functional compensation rather than a direct assessment of collateral conductance [15,21].

SPECT is the most widely used myocardial perfusion technique and remains practical for detecting stress-induced ischemia and fixed defects in clinical workflows [22]. Its broad availability and established prognostic literature are advantages. Relative, rather than absolute, perfusion assessment, lower spatial resolution, attenuation artifacts, radiation exposure, and limited ability to quantify flow reserve constrain its ability to characterize collateral physiology specifically. SPECT therefore assesses the functional impact of collaterals indirectly [21,23].

Echocardiography deserves a separate place in this framework because it is accessible, portable, radiation-free, and adaptable to bedside, exercise, or pharmacological stress testing. Stress echo identifies the functional consequence of inadequate supply through inducible regional wall-motion abnormalities; contemporary protocols can also incorporate contrast-perfusion imaging and Doppler-derived coronary flow-velocity reserve in the left anterior descending artery [24]. Contrast echocardiography can assess myocardial perfusion more directly, and a focused review described it as an experimental and clinical reference method for measuring collateral blood flow [4].

Echocardiography is therefore often the most practical functional test in coronary syndromes, but conventional wall-motion imaging is an indirect and relatively late marker of flow limitation. Image quality, operator dependence, limited acoustic windows, and the availability and expertise required for contrast perfusion or coronary-flow Doppler prevent it from replacing invasive CFI when a direct physiological collateral measurement is required. Its value is greatest when the clinical objective is serial, low-burden assessment of inducible ischemia, ventricular function, and functional reserve. Stress echocardiography’s low cost, versatility, and lack of ionizing radiation support this positioning [24].

One should use the optimal assessment method according to the specific clinical question. Use Rentrop grading to describe visible collateral anatomy during angiography. Use pressure- or Doppler-derived CFI when a quantitative invasive assessment of collateral function is required and a controlled occlusion is clinically appropriate. Use stress-perfusion imaging—CMR, PET, SPECT, or contrast/stress echocardiography—when the decisive question is whether collateralized myocardium has adequate perfusion reserve, viability, and functional protection. In most clinical settings, multimodality assessment is complementary rather than competitive: anatomy identifies the conduit, while physiological and perfusion methods determine whether it is sufficient [15,16,22].

Despite remarkable advances in multimodality imaging, echocardiography remains the most accessible and versatile technique for the functional assessment in coronary syndromes.

## 5. Echocardiographic Assessment of Coronary Collateral Circulation

### 5.1. Conventional Echocardiography

Conventional transthoracic echocardiography (TTE) does not directly visualize the coronary collateral network in routine clinical practice; its role is to characterize the myocardial functional and structural consequences of collateral-dependent perfusion. Echocardiographic assessment encompasses regional wall motion, global and regional systolic function, left ventricular (LV) remodeling, diastolic function, and right ventricular (RV) performance.

#### 5.1.1. Regional Wall Motion Abnormalities and Wall Motion Score Index

Regional wall motion abnormalities (RWMA) represent an important echocardiographic marker of the functional consequences of impaired myocardial perfusion. Several studies have demonstrated an association between the presence and functional significance of coronary collaterals and preservation of regional myocardial function [25,26,27]. Although RWMA on resting TTE can be used for the detection of CAD, relatively few studies have specifically investigated the influence of coronary collateral circulation on the diagnostic performance of RWMA, and the available evidence remains limited and somewhat conflicting. Previous studies have suggested that well-developed coronary collateral circulation may attenuate myocardial damage and reduce the likelihood of developing RWMA despite significant CAD. In contrast, Woods et al. found no significant difference in the prevalence of RWMA between patients with CAD with and without collateral circulation, and collateral-adjusted interpretation did not significantly affect the sensitivity or specificity of resting TTE for the detection of CAD [28].

#### 5.1.2. Left Ventricular Systolic Function

The relationship between CCC and global LV systolic function has been investigated iifferent clinical settings. In general, more effective collateral circulation is associated with better preservation of myocardial function, although the relationship is more consistently demonstrated for regional than for global parameters [26,27,29]. The functional significance of collateralization may be particularly evident during stress or in the setting of acute coronary occlusion. Ischemic patients with well-developed collateral circulation have demonstrated a smaller decline in LVEF during exercise compared with patients with poor collateralization [26]. Similarly, early recruitable coronary collaterals have been associated with better LV systolic recovery and preservation of myocardial viability in patients presenting late after myocardial infarction [30].

However, resting LVEF may underestimate the functional significance of CCC. In patients with coronary disease, regional myocardial dysfunction may be partially compensated by hypercontractility of non-ischemic myocardial segments, thereby maintaining a relatively preserved global LVEF despite substantial regional impairment [27]. In addition, studies using dobutamine stress echocardiography have demonstrated that good collateralization may be associated with greater myocardial viability even in the absence of significant differences in LVEF or wall motion score index (WMSI) [31]. Consequently, preserved resting LVEF should not be interpreted as evidence of adequate collateral-dependent myocardial perfusion.

#### 5.1.3. Left Ventricular Remodeling and Volumes

The extent of coronary collateral perfusion may influence post-ischemic LV remodeling by limiting myocardial injury and promoting functional recovery. In a prospective study of 70 patients with a first acute myocardial infarction successfully treated by primary angioplasty, Lee et al. demonstrated that patients with sufficient collateral flow, defined by a pressure-derived fractional collateral flow index (PDCF) >24%, exhibited more favorable LV recovery at one-month follow-up. Compared with patients with insufficient collateral flow, these patients had a significantly higher LVEF, as well as lower LV end-systolic volume index and LV end-diastolic volume index. Moreover, PDCF was the strongest predictor of regional wall-motion recovery at one month, supporting a potential protective role of functionally relevant collateral circulation in post-infarction LV remodeling [32].

The potential contribution of coronary collaterals to limiting ischemic LV dysfunction is further supported by physiological studies demonstrating a persistent “scaffold effect” of collateral circulation during coronary occlusion [33]. By maintaining myocardial perfusion during periods of reduced epicardial coronary flow, collateral circulation may limit the extent of regional dysfunction and thereby attenuate adverse ventricular remodeling.

However, structural remodeling develops progressively following myocardial injury, and differences in global LV volumes may not be consistently detectable during the early stages after infarction. Serial assessment of LV volumes, LVEF, and regional function may therefore provide greater information regarding the long-term structural consequences of inadequate collateral perfusion [33].

#### 5.1.4. Diastolic Dysfunction

Diastolic dysfunction represents an early mechanical stage of the classical ischemic cascade, occurring after perfusion and metabolic abnormalities but generally before regional systolic dysfunction, electrocardiographic changes, and angina. Functional coronary collateralization appears to influence this response. During transient coronary occlusion, collateral flow has been shown to correlate with regional diastolic parameters, including early diastolic myocardial velocity and regional isovolumetric relaxation time [34]. These findings indicate that the adequacy of collateral flow influences both systolic and diastolic myocardial responses to acute ischemia.

The relationship between CCC and diastolic function also involves LV filling pressures. A reciprocal relationship has been demonstrated between LV filling pressure and the recruitable human coronary collateral circulation, with higher LV filling pressures associated with reduced collateral recruitment [35]. This suggests a potentially bidirectional interaction between ventricular loading conditions and the functional capacity of the coronary collateral circulation.

Coronary microvascular dysfunction (CMD) may provide an additional link between impaired myocardial perfusion and LV diastolic dysfunction. Studies have demonstrated associations between CMD and preclinical diastolic dysfunction, as well as adverse LV remodeling and increased myocardial stiffness [36]. In patients with multiple cardiovascular comorbidities, CMD, oxidative stress, and myocardial stiffening may coexist and contribute to LV diastolic dysfunction [37]. Nevertheless, CMD and epicardial coronary collateralization represent distinct pathophysiological processes and should not be considered interchangeable.

#### 5.1.5. Right Ventricular Function

RV function is particularly relevant in patients with right coronary artery (RCA) disease because the RCA commonly provides the dominant blood supply to the RV myocardium. In patients with proximal RCA chronic total occlusion (CTO), impairment of RV function has been demonstrated using conventional and tissue Doppler echocardiographic parameters, including reduced tricuspid annular plane systolic excursion (TAPSE), reduced RV fractional area change (RVFAC), and increased myocardial performance index (MPI). The extent of RV dysfunction was related to the characteristics of the collateral circulation, supporting a potential role of CCC in preserving RV function in chronic RCA occlusion [38].

RV dysfunction may also occur in acute myocardial infarction even in the absence of overt RV infarction. In patients with acute MI, subtle RV impairment has been demonstrated using both conventional echocardiographic parameters and RV longitudinal strain [39]. RV echocardiographic parameters have also been associated with clinical outcomes following anterior ST elevation myocardial infarction (STEMI)and may contribute to risk stratification after reperfusion [40].

Changes in RV function have additionally been investigated following coronary revascularization. Coronary artery bypass grafting has been associated with changes in conventional and tissue Doppler-derived measures of RV function. However, these data do not directly establish a relationship between coronary collateral circulation and RV recovery and should therefore be interpreted as indirect evidence regarding the sensitivity of RV function to changes in coronary perfusion.

### 5.2. Stress Echocardiography and Functional Collateral Assessment

Stress echocardiography evaluates the functional consequences of collateral-dependent perfusion by assessing inducible ischemia and contractile reserve. Collaterals may maintain resting myocardial function despite severe coronary stenosis or occlusion, yet provide insufficient perfusion reserve during exercise or pharmacological stress.

Exercise or high-dose dobutamine stress echocardiography identifies inducible dysfunction through new or worsening regional wall-motion abnormalities, changes in wall-motion score index and LVEF, transient ischemic LV dilatation, or failure of systolic function to augment. In 212 patients with significant CAD, Chaudhry et al. demonstrated that collaterals did not prevent stress-induced ischemia. Patients whose collateral donor artery contained a flow-limiting stenosis had more extensive ischemia and worse LV function, highlighting the importance of donor-vessel hemodynamics when interpreting collateral protection [41].

Low-dose dobutamine provides complementary information on myocardial viability. Improved regional systolic thickening indicates contractile reserve, whereas a biphasic response—initial improvement followed by deterioration at higher doses—suggests viable but ischemia-prone myocardium. Sustained improvement from low- to high-dose dobutamine indicates viable myocardium, often reflecting myocardial hibernation, and suggests potential for functional recovery without independently establishing a survival benefit from revascularization.

Kumbasar et al. reported an association between well-developed angiographic collaterals and myocardial viability assessed by dobutamine echocardiography [42]. Although the authors suggested that collateral assessment could guide revascularization without additional viability testing, this historical interpretation should be viewed in the context of a small observational study. Angiographically visible collaterals alone do not establish adequate perfusion reserve, myocardial viability, or a prognostic benefit from revascularization. Subsequent stress-CMR evidence further demonstrates that collateral presence and extent do not reliably predict viability or ischemia in CTO [43]. Neither visible collaterals nor demonstrated viability alone establishes a prognostic benefit from revascularization.

Ultrasound-enhancing agents improve endocardial border delineation, while dedicated myocardial contrast imaging can additionally assess tissue perfusion. Stress MCE may identify perfusion abnormalities before wall-motion changes and characterize impaired myocardial perfusion reserve. Contrast enhancement is particularly useful when endocardial visualization is suboptimal, and perfusion assessment may provide additional information where appropriate expertise is available [44]. Nevertheless, dedicated validation of stress-perfusion findings against invasive CFI remains limited.

Earlier observations by Stone et al. suggested that preserved stress wall motion despite severe coronary stenosis could reflect effective collateral support [45]. Such findings are not specific: a negative stress examination may also reflect limited workload, submaximal heart rate, or insufficient test sensitivity. Conversely, nuclear perfusion studies have demonstrated stress-induced perfusion defects despite Rentrop grade 3 collaterals [46]. These observations distinguish preservation of resting function from adequate stress reserve.

Unlike exercise and dobutamine, which primarily increase myocardial oxygen demand, adenosine and dipyridamole assess vasodilator reserve and may provoke flow redistribution or coronary steal. In patients with major coronary artery occlusion, well-developed angiographic collaterals were associated with greater susceptibility to dipyridamole-induced wall-motion abnormalities and LV dysfunction [47]. Adenosine combined with transthoracic coronary Doppler can assess collateral flow-velocity reserve and identify a reduction in collateral-dependent flow during hyperemia [48].

Overall, stress echocardiography provides an indirect assessment of collateral-related myocardial protection rather than a measure of collateral anatomy or flow itself. Interpretation should integrate the stress protocol, donor-vessel disease, coronary anatomy, and available perfusion or physiological measurements.

### 5.3. Myocardial Contrast Echocardiography

Myocardial contrast echocardiography (MCE) was initially performed in experimental and catheterization laboratories using sonicated radiographic contrast media containing microbubbles injected directly into the coronary arteries. These early studies demonstrated that the injection route and site influenced the observed distribution of myocardial contrast. Whereas coronary angiography depicts visible epicardial collateral vessels, MCE evaluates their downstream physiological consequences by characterizing the magnitude, spatial distribution, and transmural extent of microvascular perfusion in collateral-dependent myocardium [4].

The development of commercially available ultrasound-enhancing agents enabled intravenous MCE administered by bolus or continuous infusion. These agents contain microbubbles, generally smaller than erythrocytes, that remain within the vascular compartment and exhibit rheological behavior similar to that of red blood cells [49]. During low-mechanical-index imaging, a brief high-mechanical-index impulse destroys microbubbles within the myocardium, after which their replenishment can be assessed. Replenishment curves are commonly described as:

I(t) = A(1 − e^−ßt^)

where I(t) denotes myocardial acoustic intensity at time t after microbubble destruction; A is the plateau acoustic intensity, which approximates myocardial blood volume; and β is the replenishment rate constant, which reflects microvascular blood velocity. The product A × β provides an index proportional to myocardial blood flow; absolute quantification requires appropriate calibration and standardization [4].

In chronic total coronary occlusion (CTO), homogeneous or near-normal contrast replenishment distal to the occlusion suggests effective collateral-dependent perfusion, whereas reduced or delayed replenishment indicates inadequate collateral supply despite angiographically visible collateral channels. Cho et al. examined the relationship between quantitative MCE parameters and angiographic collateral grades in patients with CTO and evaluated changes in microvascular perfusion following revascularization. Their findings suggested that quantitative MCE can characterize microvascular perfusion in CTO territories and demonstrated an association between MCE parameters and Rentrop collateral grade [50].

Detectable myocardial perfusion indicates preserved microvascular integrity and supports the presence of viable myocardium with the potential for functional recovery after revascularization. Nevertheless, preserved perfusion is not synonymous with preserved contractility, as collateral-dependent myocardium may remain viable but chronically dysfunctional [51]. Moreover, the STICH viability substudy and REVIVED-BCIS2 trial showed that viability testing did not reliably identify patients who derived a differential prognostic benefit from revascularization compared with guideline-directed medical therapy [52,53]. Thus, viability should be interpreted in the context of ischemic burden, coronary anatomy, symptoms, ventricular remodeling, and procedural feasibility.

During stress echocardiography, ultrasound-enhancing agents are recommended to improve endocardial border definition when at least two contiguous LV segments are inadequately visualized. MCE perfusion imaging can provide additional information by identifying stress-induced microvascular perfusion abnormalities, although its availability, regulatory status, and degree of standardization vary [24]. This application is particularly relevant to collateral-dependent myocardium because well-developed collaterals may preserve resting perfusion while lacking sufficient hyperemic reserve, thereby permitting inducible ischemia during stress.

In acute coronary occlusion and myocardial infarction, MCE can characterize absent tissue perfusion, residual collateral-derived perfusion before reperfusion, and persistent microvascular obstruction despite restoration of epicardial coronary flow [54,55]. After primary PCI for STEMI, MCE can assess microvascular reperfusion and identify no-reflow, providing information complementary to angiographic flow and conventional wall-motion analysis [56]. A systematic review and meta-analysis also suggested that MCE-derived viability and perfusion findings may contribute to long-term risk stratification following acute myocardial infarction [57].

MCE has also been investigated as a quantitative method for assessing collateral-derived myocardial perfusion. In an angiographically controlled study, Vogel et al. demonstrated the feasibility of measuring collateral-derived myocardial blood flow by MCE during transient coronary occlusion and validated pressure-derived collateral flow index against tissue-level perfusion measurements [3]. However, agreement among the available methods is inherently imperfect because they interrogate complementary components of collateral circulation: the Rentrop score reflects angiographic filling of the recipient epicardial artery, CFI reflects distal collateral pressure or flow, and MCE evaluates tissue-level microvascular perfusion.

In conclusion, MCE extends collateral assessment beyond angiographic visualization by evaluating the delivery of blood to collateral-dependent myocardium. It can identify preserved resting perfusion, myocardial viability, impaired hyperemic reserve, and perfusion redistribution during stress. Nevertheless, contemporary collateral-specific evidence remains limited, and standardized quantitative protocols validated against invasive CFI and absolute perfusion imaging are required before MCE can be routinely incorporated into clinical collateral assessment.

### 5.4. Deformation Imaging Echocardiography

Deformation imaging, particularly speckle-tracking echocardiography, offers a sensitive means of characterizing the mechanical consequences of collateral-dependent perfusion, detecting subtle myocardial dysfunction that may remain unrecognized by conventional wall-motion analysis or left ventricular ejection fraction. Because longitudinally oriented fibers predominate in the subendocardium—the myocardial layer most susceptible to ischemia—reduced perfusion may impair longitudinal deformation before changes become apparent in LVEF or visually assessed regional wall motion [58]. In 69 patients with successful single-vessel CTO PCI, patients with poor collaterals had lower baseline LVEF and more impaired global longitudinal strain (GLS); GLS improved significantly after PCI in the poor-collateral group; LVEF did not change significantly in either group. This supports two important ideas that well-developed collaterals may attenuate baseline deformation abnormalities and that poorly collateralized myocardium may have greater potential for mechanical recovery after restoring antegrade flow [59]. In addition, territory-based regional longitudinal strain may provide more specific information on the mechanical consequences of CTO and collateral dependence than global GLS, as localized improvement within the CTO or donor-artery territories may be attenuated when averaged across the entire left ventricle, as demonstrated by Sotomi et al. in 37 patients with CTO assessing separately regional longitudinal strain in the CTO-related territory, the collateral donor-artery territory and in the non-CTO/non-donor territory [60].

Stress speckle tracking echocardiography (STE) may reveal impaired contractile reserve or coronary steal in collateral-dependent territories, especially in cases with poorly developed coronary collaterals which are more prone than well-developed collaterals to withdrawing their blood support to the collateralized myocardium due to higher collateral pathway resistance and lower vasodilatory reserve of the collateral bed. Potential findings include: failure of regional strain to become more negative, deterioration of strain at peak stress, increased post-systolic shortening, prolongation of time to peak strain, increased mechanical dispersion. A major knowledge gap is the lack of large studies comparing stress-induced regional strain changes with Rentrop grade, Werner collateral connection grade and invasive CFI.

In acute coronary syndromes (ACS), deformation imaging detects subclinical global and regional dysfunction, characterizes the transmural extent of myocardial injury and provides prognostic information beyond LVEF. In principle, effective collateral supply may attenuate regional and particularly subendocardial deformation abnormalities by preserving tissue-level perfusion. However, contemporary evidence directly relating GLS, territorial strain or layer-specific deformation to angiographically or physiologically quantified collateral function in ACS remains scarce. Most collateral-specific deformation data derive from CTO cohorts, and extrapolation to acute coronary occlusion should therefore be cautious. Despite evidence supporting that resting GLS determined by 2D STE is a marker of severe CAD in suspected non ST elevation acute coronary syndromes (NSTE-ACS) with potentially preserved conventional systolic function [61] or that 3D STE is valuable for improving the detection of acute coronary occlusion in patients with NSTE ACS [62], there is not a robust contemporary study directly comparing myocardial deformation in ACS patients with good versus poor Rentrop-graded collaterals to date. Kim et al. performed a layer-specific strain analysis with speckle-tracking echocardiography to investigate the transmural epi-endocardial difference in myocardial damage as a predicting factor for the viability of damaged myocardium in patients with ST segment elevation myocardial infarction (STEMI) supporting the hypothesis that effective collateral perfusion might attenuate endocardial injury and preserve the normal transmural strain gradient, whereas inadequate collaterals may be associated with a more homogeneous, transmural loss of deformation [63].

Although longitudinal strain remains the most studied parameter in ischemic heart disease, circumferential and radial strain may provide complementary information on injury extent. Preserved circumferential strain despite impaired longitudinal strain may suggest subendocardial involvement, whereas impairment of both may indicate more extensive damage. Radial strain is less reproducible and more image-dependent [64]. Whether collateral supply preserves these mechanics remains unclear.

### 5.5. Myocardial Work

Myocardial work (MW) may overcome the limitations of strain analysis by integrating myocardial deformation with an estimated left ventricular pressure curve, thereby accounting for the influence of afterload [65]. The principal parameters include global work index (GWI), global constructive work (GCW), global wasted work (GWW), and global work efficiency (GWE) [66]. In coronary artery disease, ischemic myocardium typically exhibits reduced constructive work and efficiency with increased wasted work, sometimes before abnormalities in LVEF or resting wall motion become apparent [67]. Regional myocardial work may be particularly useful especially when ischemia is limited to a single coronary territory. In a study directed by Guo at al. in patients with coronary artery disease, especially with single-vessel stenosis, the regional MW indices exhibited a good diagnostic value in detecting significant myocardial ischemia compared to the standard fractional flow reserve (FFR) approach [68].

Theoretically, in collateral-dependent myocardium, preserved regional work at rest could indicate that collateral flow is sufficient to maintain mechanical energetics, whereas reduced regional GWI or GWE and increased GWW may suggest inadequate collateral support or persistent ischemia. Assessment during stress may further reveal limited collateral flow reserve despite preserved resting function [69]. However, direct evidence linking myocardial work parameters to angiographic collateral grades or invasive collateral flow index remains scarce. Therefore, myocardial work could currently be regarded as a promising complementary marker of the mechanical consequences of collateral perfusion rather than a validated measure of collateral function.

### 5.6. Three-Dimensional Echocardiography

Three-dimensional echocardiography may complement collateral assessment by providing accurate LV volumes and a volumetric evaluation of regional function without geometric assumptions [70]. Three-dimensional echocardiography segmental volume analysis can serve as a quantitative and objective tool for regional wall motion assessment in patients with ischemic heart disease [71]. Moreover, the combination of 3DE with contrast enhancement or stress imaging could improve delineation of regional wall motion and perfusion distribution. Three-dimensional speckle tracking may further characterize multidirectional deformation within collateral-dependent territories. However, direct validation against angiographic collateral grade or invasive collateral flow index is lacking, and limited temporal resolution and image-quality dependence currently restrict its clinical application.

Different echocardiographic techniques provide complementary aspects on collateral flow, myocardial perfusion, and regional function (Table 1).

## 6. Clinical Applications

### 6.1. Acute Coronary Syndromes

Echocardiography is essential in ACS for assessing left ventricular ejection fraction and detecting regional wall-motion abnormalities, while also identifying mechanical complications and alternative causes of acute chest pain [72]. Pre-existing coronary collaterals may limit infarct size and preserve regional and global left ventricular function following acute coronary occlusion. Accordingly, relative preservation of wall motion or mocardial deformation within the jeopardized territory may suggest residual collateral support; however, this finding is indirect and should be interpreted together with angiographic and perfusion data. The protective effect of coronary collaterals in patients with first acute myocardial infarction (AMI) is underlined in a MRI based study where collateral flow to the infarct-related artery was associated with more salvaged myocardium at 12 weeks, translating into greater improvement of systolic left ventricular function [73]. Collaterals may also protect the coronary microcirculation: well-developed collaterals have been associated with less microvascular obstruction after primary PCI and a strong trend toward a lower rate of death or non-fatal reinfarction [74].

In NSTEMI, coronary collaterals may attenuate regional wall-motion and deformation abnormalities despite acute culprit-artery occlusion, potentially masking the extent of myocardium at risk. Conversely, impaired territorial strain or myocardial work may help identify an occluded artery and support early invasive assessment [75]. After clinical stabilization, stress echocardiography—with perfusion imaging when available—may reveal residual ischemia when collateral flow is sufficient at rest but inadequate during increased myocardial demand [4]. Dobutamine stress echocardiography for inducible ischemia should be avoided in patients with ongoing unstable angina or active acute coronary syndrome, decompensated heart failure, uncontrolled clinically significant arrhythmias, severe uncontrolled hypertension, or haemodynamically significant LV outflow tract obstruction. Other contraindications include acute myocarditis or pericarditis, acute pulmonary embolism, and acute aortic dissection [24].

Therefore, integrating resting and stress-induced wall motion, deformation and perfusion findings may contribute to risk stratification by distinguishing effective resting collateral support from limited collateral flow reserve [24].

### 6.2. Chronic Coronary Syndrome

In chronic coronary syndromes, the clinical significance of collateral circulation depends on the balance between underlying disease burden and the functional protection provided by collateral perfusion. As discussed in Section 3, the findings of Koerselman et al. illustrate why angiographically visible collaterals should not be interpreted as an independent marker of favorable prognosis [14]. Assessment should therefore consider collateral-dependent myocardial function and perfusion alongside coronary anatomy and the patient’s clinical risk.

Abbott et al. compared the baseline characteristics and cumulative one-year event rates of 6183 consecutive patients undergoing PCI by target vessel collateral status, angiographically defined as absent (5051 subjects), treated artery supplied collaterals (239 subjects) and treated artery received collaterals (893 subjects). The group that received percutaneous coronary intervention on a collateral receiving artery had lower adjusted death or myocardial infarction rates whereas cumulative one-year mortality was 4.7% in the group without and 4.1% in the group with collaterals to the vessel undergoing PCI. Due to the limitations of this study, the important all-cause mortality data are difficult to interpret [76].

The prevalence of chronic total occlusions in patients with CAD vary between 20 and 30%, these patients usually present with stable angina, unless other coronary lesions progress and lead to acute coronary syndromes. Moreover, their risk is higher if a collateral supplying artery is involved in an acute event or during a percutaneous procedure [77].

Coronary collaterals in chronic total occlusion (CTO) carry an essential role in the perfusion of the myocardium, considering that in the case of a CTO, myocardial perfusion relies solely on the collaterals and microvascular resistance. Assessing collateral function in this situation is fundamentally different in clinical practice [77].

Randomized CTO trials should be interpreted according to their specific endpoints rather than summarized as uniformly showing no reduction in major adverse cardiovascular events [78,79]. At three years, EuroCTO found no statistically significant difference in cardiovascular death or non-fatal myocardial infarction between optimal medical therapy and PCI (3.7% versus 6.2%; *p* = 0.29). However, MACE occurred more frequently with optimal medical therapy than with PCI (21.2% versus 11.2%), largely because of ischemia-driven revascularization [79]. These findings distinguish the effect on subsequent revascularization from an established reduction in death or myocardial infarction. Selection for CTO revascularization should integrate symptoms despite medical therapy, ischemic burden, scar extent and residual viability, coronary anatomy, procedural feasibility and risk, and the clinical setting. Viability assessment may help characterize the potential for functional recovery, but viable myocardium alone does not establish that revascularization will confer a prognostic benefit. Similarly, angiographically visible collaterals should not independently determine the decision to revascularize.

## 7. Integration with Multimodality Imaging

Echocardiography provides an accessible and non-invasive functional assessment of the myocardial consequences of coronary collateral circulation (CCC), but its findings should be interpreted within a multimodality imaging framework. Conventional and deformation imaging assess regional myocardial function, while stress echocardiography and myocardial contrast echocardiography can identify inducible ischemia and impaired perfusion reserve, CMR, PET/SPECT, and CCTA provide complementary information on tissue viability, perfusion reserve, and coronary anatomy [80]. Their integration may therefore better characterize the functional effectiveness and myocardial impact of collateral flow.

CMR provides important complementary information to echocardiography by combining accurate assessment of ventricular function with tissue characterization and myocardial perfusion. Late gadolinium enhancement allows quantification of infarct size and transmurality, while CMR can also identify microvascular obstruction and, during stress, residual ischemia [44]. In collateralized territories, these parameters may help determine whether preserved regional mechanics on echocardiography reflect effective myocardial protection and viability or coexist with irreversible injury or persistent ischemia [81]. Thus, integration of echocardiographic deformation indices with CMR may provide a more comprehensive assessment of the myocardial consequences of collateral flow.

Nuclear and CT imaging provide additional functional and anatomical information. PET enables absolute quantification of myocardial blood flow and myocardial flow reserve, whereas SPECT evaluates the extent and severity of regional perfusion abnormalities. These techniques may therefore identify inadequate perfusion reserve in collateral-dependent myocardium despite preserved resting function [82]. Conversely, CCTA provides detailed characterization of coronary anatomy and, in selected cases, visualization of collateral pathways [44]. Combining anatomical information from CCTA or invasive angiography with myocardial mechanics from echocardiography and perfusion/tissue characterization from PET, SPECT, or CMR may better define the anatomical presence, functional effectiveness, and myocardial impact of coronary collateral circulation.

Modality selection should also consider contraindications to the examination, contrast agent, and stressor separately. Resting TTE has few contraindications, although poor acoustic windows may limit interpretation; ultrasound-enhancing agents are contraindicated in patients with known hypersensitivity to the selected agent or its components. For CCTA and invasive angiography, previous severe reactions to iodinated contrast and acute kidney injury or advanced renal dysfunction require individualized risk assessment. CMR requires screening for unsafe implants or metallic foreign bodies; severe claustrophobia may limit feasibility, while gadolinium administration requires separate consideration in patients with severe renal dysfunction. PET, SPECT, and CT involve ionizing radiation and are generally avoided during pregnancy unless clinically justified.

Pharmacological stress introduces additional restrictions. Adenosine and dipyridamole should be avoided in patients with active bronchospasm, including asthma or COPD with ongoing wheezing, significant hypotension, or high-grade atrioventricular block without a functioning pacemaker. Regadenoson may be considered in selected patients with stable airway disease but should also be avoided during active bronchospasm or acute respiratory exacerbation. Recent caffeine or methylxanthine exposure may reduce vasodilator efficacy. Dobutamine is unsuitable in acute coronary syndromes, uncontrolled significant arrhythmias, severe uncontrolled hypertension, or hemodynamically significant LV outflow tract obstruction. Thus, contraindications to a stress agent do not necessarily preclude the imaging modality itself, and an alternative protocol may be appropriate.

Multimodality imaging integrates complementary anatomical and functional information, enabling a more comprehensive assessment of coronary collaterals and their effects on myocardial function (Table 2).

## 8. Future Perspectives

Future assessment of coronary collateral circulation may benefit from advanced echocardiographic techniques capable of detecting subtle changes in myocardial mechanics that remain undetected by conventional LVEF and wall-motion analysis. Layer-specific and regional strain may provide a more sensitive characterization of ischemia-related deformation, particularly in the vulnerable subendocardial layer, while myocardial work integrates deformation with loading conditions and may offer additional information on regional myocardial performance and efficiency. Recent studies suggest that using layer-specific strain and myocardial work techniques may improve the diagnostic performance of dobutamine stress echocardiography or treadmill exercise test for coronary ischemia in patients with preserved resting wall motion [83,84]. Despite that the 2026 ASE/EACVI strain consensus does not currently endorse layer-specific strain as superior to full-wall strain for routine clinical use [85], future studies should determine whether regional patterns of preserved strain and myocardial work within collateral-dependent territories can serve as non-invasive markers of functionally effective collateral flow and whether their integration with three-dimensional deformation analysis can improve characterization of the myocardial protective effects of coronary collaterals.

Advances in myocardial contrast echocardiography (MCE) may enable a more quantitative assessment of collateral-dependent myocardial perfusion. Unlike coronary angiography, which primarily depicts the anatomical extent of collateral vessels, MCE interrogates the myocardial microcirculation and can estimate myocardial blood flow from microbubble replenishment kinetics, integrating myocardial blood volume and microvascular flow velocity. Quantification at rest and during vasodilator stress may additionally provide estimates of myocardial perfusion reserve, potentially allowing assessment of whether collateral flow is sufficient not only to maintain resting perfusion but also to meet increased myocardial demand [86]. Recent developments in automated quantitative analysis including automated strain and quantitative myocardial contrast echocardiography (MCE) with dedicated software further support the feasibility of deriving perfusion and flow-reserve parameters from advanced echocardiographic methods, exhibiting the potential to be a clinical alternative to cardiac PET [87]. Future studies specifically comparing these quantitative perfusion indices with invasive collateral flow index and angiographic collateral grading could establish MCE as a non-invasive approach for distinguishing anatomically visible collaterals from functionally effective collateral networks.

Future evaluation of coronary collateral circulation should increasingly integrate advanced echocardiographic markers with invasive physiological measurements of collateral function. Angiographic grading systems, such as the Rentrop score, provide a semiquantitative assessment of collateral vessel filling but are influenced by hemodynamic and technical factors and do not directly quantify the functional capacity of the collateral network. In contrast, pressure- or Doppler-derived collateral flow index (CFI) provides a quantitative estimate of collateral blood supply during coronary occlusion and remains an important physiological reference for functional collateral assessment [86]. Future prospective studies correlating CFI and intracoronary pressure or flow measurements with regional strain, myocardial work, stress-induced deformation and quantitative myocardial perfusion could identify non-invasive echocardiographic markers of functionally effective collateralization. Such an integrated approach may ultimately bridge the gap between anatomical visualization of collateral vessels and assessment of their ability to preserve myocardial perfusion and contractile function.

Studies should move beyond the cross-sectional evaluation of coronary collateral circulation toward longitudinal assessment of its functional myocardial consequences. Serial echocardiography, particularly using speckle-tracking techniques, could characterize changes in global and regional myocardial mechanics following acute coronary syndromes, CTO revascularization, or other therapeutic interventions. Such an approach may help determine whether preserved or improving deformation in collateral-dependent territories reflects sustained myocardial protection and could clarify its relationship with functional recovery, reverse remodeling, residual ischemia, and subsequent clinical outcomes. Recent data showing changes in longitudinal strain after CTO revascularization according to the degree of collateral supply support the potential value of serial deformation imaging for monitoring the dynamic interaction between collateral circulation and myocardial function [88].

Artificial intelligence may provide new opportunities for a more comprehensive and objective characterization of coronary collateral function. Machine-learning models can integrate conventional echocardiographic parameters with regional strain, myocardial work, perfusion indices, clinical characteristics, and angiographic or physiological data, potentially identifying complex imaging characteristics associated with functionally effective collateralization. Guo et al. prospectively studied patients undergoing coronary angiography and developed an machine learning model integrating 59 echocardiographic features, including myocardial work, strain, mechanical dispersion and LA strain, demonstrating that machine-learning models can improve the echocardiographic detection of coronary artery disease, while radiomic analysis of myocardial contrast echocardiography can extract quantitative image features associated with subsequent myocardial functional recovery after revascularization [89,90]. These approaches may be particularly relevant to coronary collateral assessment, where myocardial protection is heterogeneous and strongly dependent on the vascular territory involved. Future development of automated segmental analysis combining mechanics and perfusion with invasive collateral measurements may facilitate identification of imaging phenotypes reflecting effective collateral flow and potentially improve prediction of myocardial recovery and clinical outcomes.

Future collateral evaluation should shift from anatomical grading toward functional and biological characterization reflecting angiogenesis, arteriogenesis, endothelial function, and inflammation. In particular, microRNA signatures may provide complementary information on the biological mechanisms underlying collateral vessel development and functional maturation. Recent studies have identified specific circulating and transcoronary microRNA profiles associated with collateral development and collateral flow index, supporting their potential role as biomarkers of functional collateral capacity [91,92].] In 126 CTO patients transcoronary gradients of several miRNAs—including miR-21, miR-26a, miR-92a and miR-126—were associated with collateral development, moreover miR-26a and miR-21 showed particularly strong positive correlations with CFI [91,92]. Combining such molecular data with echocardiographic measures of regional deformation, myocardial work, and perfusion could provide a more comprehensive phenotype of collateral-dependent myocardium. This integrated approach may ultimately help distinguish the anatomical presence of collateral vessels from biologically active and functionally effective collateral networks capable of preserving myocardial perfusion, mechanics, and viability (Figure 1).

The principal studies informing collateral assessment are compared in Table 3, highlighting differences in study design, clinical setting, assessment methods, findings, and limitations.

## 9. Conclusions

Coronary collateral circulation represents an important adaptive response to coronary artery disease and echocardiography provides complementary information on the myocardial consequences of coronary collateral circulation through assessment of ventricular function, regional deformation, contractile reserve, and myocardial perfusion. These findings may help characterize collateral-dependent myocardium, but they should not be interpreted as specific measures of collateral adequacy, particularly where direct physiological validation is lacking. Invasive coronary angiography depicts angiographically visible collateral pathways, while CCTA may demonstrate selected visible collateral channels; neither delineates the entire collateral network nor independently establishes its functional adequacy. Integrating echocardiography with coronary anatomy, invasive physiology, and tissue characterization may improve assessment, although the incremental clinical value of emerging echocardiographic techniques requires further validation.

## Figures and Tables

**Figure 1:**
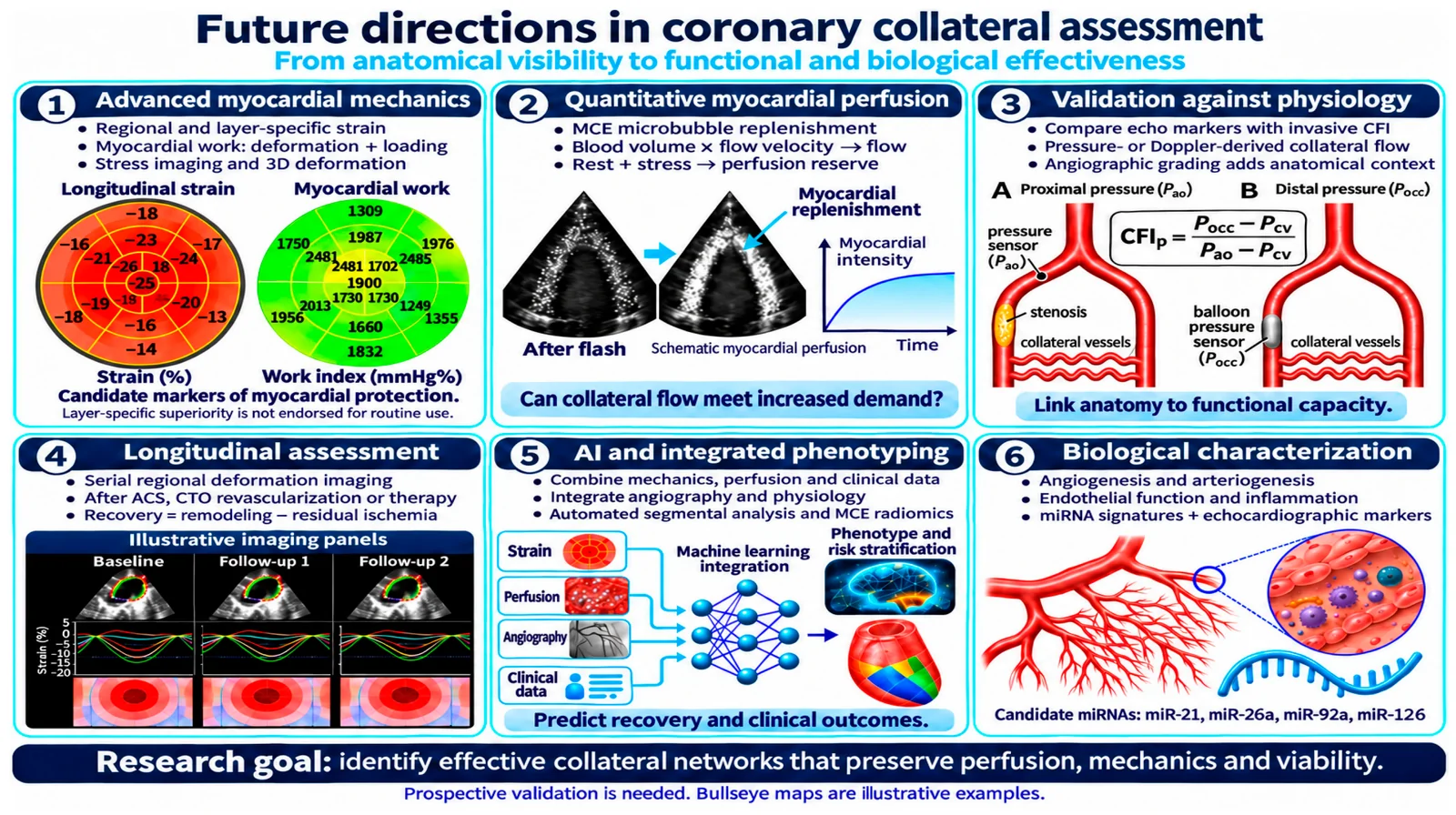
Future perspectives in assessing coronary collateral circulation.

**Table 1:** Role of different echocardiographic techniques in assessing coronary collateral circulation.

Echocardiographic Method	Role in Assessing Coronary Collateral Circulation	Main Parameters/Findings	Key Advantages	Main Limitations
**Conventional TTE**	Indirect assessment of the functional consequences of collateral-dependent perfusion	Regional wall-motion abnormalities LV systolic function chamber remodeling	Widely available, non-invasive, repeatable, bedside	cannot directly visualize most collateral vessels; limited sensitivity for subtle ischemia
**2D/3D echocardiography**	Characterizes structural and functional myocardial consequences of chronic ischemia and collateralization	LV volumes EF regional wall motion ventricular remodeling	Comprehensive ventricular assessment; 3D improves volumetric quantification	Collateral circulation is assessed indirectly
**Speckle-tracking echocardiography (STE)**	Subclinical regional myocardial dysfunction potentially related to collateral-dependent territories	GLS regional longitudinal/circumferential strain myocardial work	Sensitive to subtle dysfunction; quantitative; non-invasive; potentially useful for serial assessment	Strain is influenced by loading conditions, scar and ischemia; no direct visualization of collaterals
**Stress echocardiography**	Evaluates the functional significance of collateral circulation by assessing myocardial response to increased demand	Inducible wall-motion abnormalities contractile reserve ischemic burden	Provides dynamic functional information; no radiation; relatively accessible	Operator-dependent; collateral vessels are not directly visualized; interpretation can be difficult in multivessel disease
**Myocardial contrast echocardiography (MCE)**	Evaluates myocardial perfusion supplied by collateral vessels, particularly distal to an occlusion	Myocardial blood volume, myocardial blood flow, perfusion defects, replenishment kinetics	Real-time perfusion assessment high temporal resolution no ionizing radiation	Requires contrast and expertise; limited availability; collateral vessels are generally not directly visualized
**Contrast stress echocardiography**	Assesses collateral-dependent myocardial perfusion under stress	Inducible perfusion defects, perfusion reserve	Combines stress and perfusion assessment; potentially identifies functionally relevant collateralization	Technically demanding; limited standardization; indirect assessment

**Table 2:** Five complementary assessment domains.

Assessment Domain	Methods or Techniques	Information Provided	Interpretation in Collateral Assessment
**Anatomical imaging**	Invasive coronary angiography; CCTA in selected cases	Coronary stenosis or occlusion, visible collateral connections, and recipient-vessel filling	Describes the anatomical substrate and angiographically visible collateralization; does not directly quantify tissue perfusion or establish functional adequacy.
**Invasive physiological assessment**	Pressure-derived or Doppler-derived CFI during coronary occlusion	Quantitative indices of collateral pressure support or relative collateral flow	Provides a collateral-specific physiological assessment during occlusion.
**Myocardial tissue perfusion and perfusion reserve**	Quantitative PET perfusion; stress-perfusion CMR; SPECT perfusion; MCE at rest and during stress	Resting or stress perfusion, inducible perfusion defects; myocardial blood flow and perfusion reserve	Evaluates blood delivery to collateral-dependent myocardium. PET and SPECT perfusion findings do not directly measure collateral flow.
**Scar and myocardial viability**	CMR LGE; low-dose dobutamine echocardiography or CMR; selected SPECT viability protocols; FDG-PET with perfusion imaging	Scar extent and transmurality, contractile reserve, or preserved metabolism	Characterizes myocardial injury and residual viability. CMR LGE assesses scar, whereas stress-CMR perfusion assesses perfusion and inducible ischemia. Viability does not establish adequate collateral reserve.
**Myocardial function and mechanics**	Conventional TTE; tissue Doppler; 2D/3D echocardiography; STE; MW; stress echocardiography; cine CMR	Ventricular volumes, LVEF, regional wall motion, deformation, work indices, and contractile response to stress	Assesses myocardial consequences potentially associated with collateral support. Findings are influenced by scar, loading conditions, reperfusion, and other determinants and are not specific measurements of collateral function.

**Table 3:** Comparative overview of principal studies informing the assessment and clinical significance of coronary collateral circulation.

Study	Design and Population	Clinical Setting and Assessment	Principal Finding	Main Limitations and Interpretation
**Nathoe et al., 2006 [14]**	Observational analysis of 561 patients enrolled in a randomized comparison of coronary stenting and CABG	Stable CAD undergoing revascularization; Rentrop grading; 1-year cardiac death or MI	Angiographic collaterals were associated with a lower adjusted risk of cardiac death or MI after revascularization.	Collateral status itself was not randomized; patients undergoing revascularization and do not establish the effect of collaterals in untreated CAD.
**Physiology**				
**Seiler et al., 2002 [34]**	Within-patient physiological study; 50 CAD patients without previous MI	Elective angioplasty; pressure-derived CFI and tissue Doppler before and during coronary occlusion	Collateral flow was related to preservation of regional systolic and diastolic function during occlusion.	Brief, controlled occlusion in selected patients does not reproduce prolonged spontaneous
**Hoole et al., 2012 [33]**	Mechanistic physiological study; 25 patients with normal LV function awaiting PCI	Controlled coronary occlusion; collateral assessment and invasive LV mechanics	Supported a collateral-related “scaffold” effect and limitation of ischemic LV dysfunction.	Small, selected population with preserved baseline function;
**Stress imaging. Viability**				
**Chaudhry et al., 2004 [41]**	Observational comparative study; 212 consecutive patients with significant CAD	Stress echocardiography and angiography; groups defined by collateral presence and donor-vessel stenosis	Collaterals did not prevent stress-induced ischemia; jeopardized donor vessels were associated with greater ischemic burden and worse LV function.	Groups differed in disease burden and baseline function. Angiographic assessment and an interval of up to 3 months between tests limit direct physiological attribution.
**Kumbasar et al., 2007 [42]**	Prospective observational study; 47 patients, each with one totally occluded coronary artery	Angiographic collateral assessment and dobutamine echocardiography	Well-developed collaterals were associated with myocardial viability assessed by contractile reserve.	Small, selected cohort; contractile reserve is a surrogate endpoint. The association does not establish that collateral grading alone can guide revascularization or predict clinical benefit.
**Aboul-Enein et al., 2004 [46]**	Comparative imaging study; 56 patients with single-vessel CTO and no previous MI	Rest–stress SPECT and angiography; Rentrop grades 1–2 versus 3	Excellent angiographic collaterals were associated with fewer resting wall-motion abnormalities but did not prevent stress perfusion defects.	Selected population without previous MI; relative SPECT perfusion rather than absolute flow measurement; investigations performed within a 6-month interval.
**Myocardial contrast echocardiography**				
**Janardhanan et al., 2004 [55]**	Serial imaging study; 70 AMI patients screened, with analysis focused on 20 patients with persistent infarct-related artery occlusion	Intravenous MCE 7–10 days after thrombolysis; contractile reserve assessed after revascularization	Preserved MCE perfusion in akinetic segments predicted subsequent contractile reserve.	Small selected subgroup and segment-based analysis; contractile reserve after revascularization is an indirect marker of preceding collateral support.)
**Vogel et al., 2006 [3]**	Physiological validation study; 30 stable CAD patients, 37 stenoses; successful MCE analysis in 32 cases	Simultaneous pressure-derived collateral assessment and quantitative MCE during and after angioplasty	Supported validation of pressure-derived collateral assessment against tissue-level collateral perfusion measured by MCE.	Small procedural cohort; incomplete imaging feasibility and controlled balloon occlusion restrict extrapolation to routine serial assessment or ACS.
**Cho et al., 2015 [50]**	Prospective observational study; 44 CTO patients	Quantitative MCE, angiographic collateral grading, and follow-up after revascularization	Baseline MCE-derived myocardial blood flow was higher with grade III than grade I–II collateral filling.	Small cohort; angiographic grading provides an anatomical comparator rather than an independent physiological reference. Post-revascularization changes also reflect restored antegrade flow.
**Deformation imaging**				
**Kurklu et al., 2024 [59]**	Serial observational study; 69 patients undergoing successful single-vessel CTO PCI	GLS and LVEF before PCI and during follow-up, according to collateral supply	Poorly collateralized patients had greater baseline impairment and showed improvement in GLS after PCI, without significant LVEF change.	Selected successfully revascularized cohort; short follow-up and baseline group differences limit causal inference. GLS recovery does not directly quantify collateral flow.
**Sotomi et al., 2017 [60]**	Prospective observational study; 37 CTO-PCI patients	Global and regional strain in CTO, donor-vessel, and remote territories before PCI, at 1 day, and at 3 months	Early strain improvement was partly attenuated over time; donor-territory regional improvement persisted at 3 months, while LVEF was unchanged.	primarily evaluates revascularization-related mechanical changes rather than validating strain against collateral flow.
**Emerging techniques**				
**Guo et al., 2022 [68]**	Retrospective single-center study; 84 patients, 92 stenotic vessels	Regional MW compared with invasive FFR in suspected CAD	Regional work indices showed diagnostic value for functionally significant stenosis, particularly in single-vessel disease.	FFR validation is not collateral-specific validation; collateral grades and CFI were not the principal reference measurements.
**Lin et al., 2022 [83]**	Retrospective single-center study; 89 patients with suspected CAD	Treadmill stress echocardiography; layer-specific GLS and GWE compared with angiographic stenosis	Peak GWE and endocardial GLS improved identification of significant CAD in patients with normal baseline wall motion.	Does not directly establish detection of collateral-mediated protection.
**Qamruddin et al., 2025 [84]**	Single-center observational proof-of-concept study; 45 analyzed patients, including 20 with abnormal DSE	Peak GLS and MW during DSE; secondary anatomical or perfusion testing in patients with abnormal DSE	Adding peak GWE improved the positive predictive value of conventional DSE for significant CAD.	Collateral-specific diagnostic accuracy was not established.

## Data Availability

No new data were created or analyzed in this study. Data sharing is not applicable to this article.

## Abbreviations

The following abbreviations are used in this manuscript:

2D	two dimensional
3D	three dimensional
ACS	acute coronary syndromes
AMI	acute myocardial infarction
ASE	American Society of Echocardiography
bFGF	basic fibroblast growth factor
CAD	Coronary artery disease
CCC	Coronary collateral circulation
CFI	collateral flow index
CMD	Coronary microvascular dysfunction
CMR	cardiac magnetic resonance
CTO	chronic total occlusion
EACVI	European Association of Cardiovascular Imaging
FFR	fractional flow reserve
FGF	fibroblast growing factor
GCW	global constructive work
GLS	global longitudinal strain
GM-CSF	macrophage colony stimulating factor (recombinant human)
GWE	global work efficiency
GWI	global work index
GWW	global wasted work
HIF-1α	Factor 1-alpha
LA	left atrium
LV	left ventricle
LVEF	left ventricular ejection fraction
MCE	Myocardial contrast echocardiography
MCP-1	monocyte chemotactic protein 1
MMPs	Matrix metalloproteinases
MPI	myocardial performance index
MW	Myocardial work
NSTE-ACS	non ST elevation acute coronary syndromes
PCI	percutanoeus coronary intervention
PDCF	pressure-derived fractional collateral flow index
PET	positron emission tomography
RCA	right coronary artery
RNA	ribonucleic acid
RV	right ventricle
RVFAC	right ventricular fractional area change
RWMA	regional wall motion abnormalities
SPECT	single photon emission computed tomography
STE	speckle tracking echocardiography
STEMI	ST elevation myocardial infarction
TAPSE	tricuspid annular plane systolic excursion
TNF-α	tumor necrosis factor alpha
TTE	transthoracic echocardiography
VEGF	vascular endothelial growth factor
WMSI	wall motion score index
